# Experiences of participation in an education and support group for carers of people with longer-term psychosis: a qualitative study

**DOI:** 10.1186/s40359-024-02288-2

**Published:** 2025-02-21

**Authors:** Isabel Millard, Jo Billings, Philippa Greenfield, Jackie Bailey, Dave Fearon, Henry J. Whittle, Helen Killaspy

**Affiliations:** 1 Division of Psychiatry, University College London, London, UK; 2 Camden and Islington NHS Foundation Trust, London, UK

**Keywords:** Qualitative research, Support group, Psychoeducation, Serious mental illness, Psychosis, Informal caring/caregivers, Family, Carers

## Abstract

**Background:**

It is well-established that carers of people with psychosis require support given the stress, social isolation and health problems commonly associated with their caring role. However, interventions for carers are under-researched and underprovided. Psychoeducation and support groups can improve carers’ experiences of caregiving, but there is insufficient evidence to inform an optimal intervention. Furthermore, the limited existing literature tends to focus on carer groups provided within early intervention services. We designed and implemented an education and support group programme for carers of people with longer term psychosis. The group ran twice, online in 2021 and face-to-face in 2023. We conducted a qualitative service evaluation to explore participants’ experiences of participation in the group and inform its further refinement.

**Methods:**

We conducted in-depth, semi-structured interviews before and after each group programme which explored carers’ experiences of caring and of group participation, in order to understand its impact, potential mechanisms of effect, and any helpful or challenging aspects. Facilitators also provided written reflective notes. Data were analysed using reflexive thematic analysis.

**Results:**

Seventeen carers and four facilitators participated. The group programme received highly positive feedback. Four main themes were identified regarding its impact on the experience of caring: 1) isolated versus connected and supported; 2) disempowered versus empowered; 3) grief, guilt, worry versus acceptance; 4) unmet needs: the challenges associated with participants’ caring roles which were not addressed by group participation. The group’s mechanisms of effect included: building connections between carers, building carers’ connections with services, and providing psychoeducation that improved carers’ confidence and increased their empathy for their relative. Key components of the group’s design were: attendees having similar experiences of caring attendees receiving clear information about the group’s purpose and format, and facilitation by senior mental professionals who enabled discussion. The main area for improvement was to increase accessibility to carers from minority groups.

**Conclusions:**

This study explored participants' experiences of a psychoeducation and support group for carers of people with longer-term psychosis and showed that it was largely acceptable and received positively by participants. The results may explain why recent trials of carer focussed interventions that have relied on digital contact were not effective. Further research is needed, including clinical trials, to investigate the clinical and cost effectiveness of face-to-face carer groups.

**Supplementary Information:**

The online version contains supplementary material available at 10.1186/s40359-024-02288-2.

## Introduction

Carers have been defined in the NICE Guideline for psychosis and schizophrenia as ‘family and friends who may or may not live with the service user, and who provide informal and regular care and support to someone with a severe mental illness (SMI) such as psychosis and schizophrenia’ [[1] p.65].

Carers play an invaluable role in supporting people with SMI and mental health services rely on their input [2]. The carer role is challenging. Carers of people with psychosis report high levels of stress, burnout and social isolation, as well as physical and mental health problems [3–6]. These difficulties can impede their ability to provide support, which may, in turn, impair the recovery of the person they care for [7, 8]. Carer support is a government and NHS priority, yet services are failing to meet this need [9, 10]. This may, at least in part, be because interventions for carers and their implementation have been under-researched [9]. Nevertheless, studies of family interventions provide useful insights.

Structured family interventions are delivered by a therapist, to one family or multifamily groups, with the patient included [11, 12]. They include psychoeducation, strategies to improve problem solving and communication skills, as well as the development of relapse prevention plans [12]. Multi-family groups also provide social connection. Family interventions have been shown to prevent relapse in psychosis, and reduce ‘carer burden’ [1, 11, 13]. However, they are poorly implemented [14]. In England, a National Clinical Audit of Psychosis in 2018 found that only 12% of patients in contact with their families had been offered a family intervention [15]. Other UK studies have found implementation rates vary between 0 and 53% [14]. Barriers to implementation of structured family interventions exist at multiple levels [14]. Organisational level barriers include a lack of senior management support and insufficient resources [14, 16–18]. Barriers at the level of individual staff include a lack of confidence in working with families, and time constraints [16, 17]. Families can also find the intervention time consuming, and some experience the presence of the service user as unhelpful [18–20]. Barriers at the level of the service user include a reluctance to engage due to reservations about family involvement in their care, and symptom severity that precludes attendance [18]. As a result, there is a call for more carer-focussed interventions [9, 10, 1]. Despite limited evidence, these interventions are recommended by NICE Guidelines for psychosis and schizophrenia and described as ‘any structured programme offered individually or in a group involving an interaction between an information provider and the carer, which has the primary aim of offering information about the condition, and the provision of support and management strategies to carers, and delivered to the carers without the service user present’ [[1] p.83]. Unlike family interventions, carer focussed interventions seek to improve outcomes for carers, rather than patients. They are also less resource intensive and can be delivered to larger numbers [18]. However, they vary widely and there is a lack of research to inform the most effective format [9, 1].

A systematic review of randomised controlled trials (RCTs) of carer interventions found very low-quality evidence to suggest that psychoeducation (8 RCTs) and support groups (3 RCTs in SE Asia) improved carers’ experiences of caregiving and reduced their psychological distress [9]. Subsequently, there have been three quasi-experimental studies investigating carer support groups in India [21], the UK [22] and Australia [23]. Although these varied in focus and number of sessions (three to seven), all included a psycho-educative component and were shown to improve both carers’ understanding of mental illness and their relationship with their relative [21–24]. However, two UK-based RCTs of digital psychoeducation and peer support interventions failed to show any advantage over passive information sources such as websites or resource directories, but the peer support intervention was limited to posting on-line forums in both studies [25, 26]. Results from qualitative studies provide additional insights into how such interventions may benefit carers. In 2009, a systematic review of four qualitative studies of support groups for carers of people with SMI found that they improved carers’ ability to cope; key components included information about the illness, as well as support and respect from peers and professionals [27]. However, the interventions were heterogenous in design and setting (three out-patient settings in Hong Kong, Belgium, Australia and one UK based inpatient setting), and the studies were generally low quality [27].

Two subsequent qualitative studies have been conducted in Australia, one with carers of patients of a community mental health team that used focus groups and a self-completed questionnaire, [28] and one with carers of patients of an early intervention for psychosis team that used in-depth interviews [19]. Both identified peer support as a key component, as well as the opportunity to discuss their experiences openly [28, 19].

Four further qualitative studies in this field have been conducted in the UK. These include three studies of carers of people receiving support from early intervention services [20, 29, 30] and one study of carers of those receiving longer term inpatient care [31]. Findings were generally congruent, despite differences between studies in the setting and number of sessions offered (from 10 to open ended): participants found carer support groups helpful for sharing ideas, information and coping strategies and valued the expert information provided [20, 29–31]. The mutual support and collective experience facilitated the expression of difficult emotions and reduced feelings of isolation, guilt and stigma [20, 29, 30]. Riley et al., also showed that carers were more able to cope with their caring role, and found the absence of the patient helpful [20]. Of note, all four studies were small and their findings may not be transferable to other carers or settings [20, 29–31].

In summary, whilst the evidence for carer support groups for people with SMI suggests they may be associated with benefits for carers, the evidence is limited to a relatively small number of studies of limited quality, evaluating heterogenous interventions in varied healthcare settings. The current evidence is therefore unable to inform an optimal intervention. In addition, as the majority of studies internationally have explored the experiences of carers of people at the earlier stages of their psychosis, further research is needed to understand the support and information needs of carers of people with longer term SMI, to inform how best to design support groups for them [31].

We therefore conducted a qualitative service evaluation of a psychoeducation and support group for carers of patients of two community mental health teams and a community mental health rehabilitation team working with people with SMI based in North London. In the absence of an established gold standard intervention for carer support, we co-produced the design of the group based on 1) a review of the relevant literature 2) input from carers and consultation with mental health professionals and 3) piloting. In keeping with the MRC guidance on the development of complex interventions, we aimed to further refine the group following evaluation [32].

The aim of this study was to conduct a qualitative evaluation of the psychoeducation and support group for carers of people with SMI. Specifically, our study objectives were to:

1. Explore the impact of attending the group on participants’ experiences of caring.
2. Gain participants’ views on the group structure, content and processes to inform its ongoing development.

## Methods

### Study design and rationale

A qualitative design, with pre- and post-group semi-structured interviews, was chosen to investigate carers’ experiences of caring and group participation, in order to understand its impact, mechanisms of effect, as well as any helpful or challenging aspects [33].

### Ethical and other approvals

This study was a service evaluation and consultation with the local NHS Trust R&D department confirmed that no ethical approval was required. All participants gave written, informed consent prior to taking part.

### Description of intervention

#### The friends, family and carers’ education and support group

The intervention comprised a closed carer psychoeducation and support group for a maximum of 16 carers, with 8 weekly sessions, from 17:30—19:30 pm. It ran twice, online using Zoom Video Communications in February–March 2021 due to the Covid-19 pandemic, and face-to-face at the team base in February–March 2023. Attendees of the 2021 group therefore required access to Zoom on a personal computer or handheld device. The first author (IM) set up and co-facilitated the group.

Each meeting consisted of an initial psychoeducational talk (Table 1) (45 min), a tea break (15 min) and a closed, small group session in which carers shared experiences (45 min, maximum eight carers per small group). The small groups were facilitated by two mental health professionals including one psychiatrist (IM or PG).

**Table 1:** Timetable of educational talks

Talks at The Friends, Family and Carers’ Education and Support Group
Week 1: Carers’ rights - the Care Act, Carers’ Assessments and The Triangle of Care
Week 2: Psychosis - symptoms, diagnosis and causes
Week 3: Medication to treat psychosis
Week 4: The Mental Health Act and crisis care
Week 5: Open Dialogue
Week 6: Cognitive behavioural therapy for psychosis
Week 7: Physical health in SMI
Week 8: Negative symptoms and the role of occupational therapy

Staff of the relevant community teams (see below ‘Setting’) were asked to invite all carers to the group.

### Setting

The study involved carers of clients of two community mental health teams and a community rehabilitation team in the London Borough of Islington, part of Camden and Islington NHS Foundation Trust. Islington is ethnically diverse with areas of high economic deprivation and a high prevalence of SMI. The three teams together have a caseload of approximately 950 service users, most of whom have a primary diagnosis of schizophrenia or other psychosis. Community mental health teams generally treat people with longer-term psychosis (ranging from three years to decades since diagnosis), following transfer from Early Intervention Services.

### Recruitment, sampling and participants

All group members were informed about the evaluation and offered the opportunity to participate. It was explained there was no obligation to take part in the study. An information sheet was provided, and written consent was obtained. As many participants as possible were recruited to optimise the depth and potential transferability of the data [34]. Inclusion and exclusion criteria are described in Table 2.

**Table 2:** Inclusion and exclusion criteria

**Inclusion Criteria**
**1. Carers who attended one of the two carer psychoeducation and support group programmes**
**2. Participants for the 2021 group required access to Zoom Video Communications on a personal computer or handheld device**
**Exclusion Criteria**
**1. Insufficient understanding of English to give informed consent or to participate in qualitative interviews**

### Data collection

#### Pre and post-group semi-structured interviews

Topic guides (Appendix 1) for the semi-structured interviews were drafted by IM and refined in consultation with the study primary supervisor (HK). Content was based on relevant themes in the literature and the researchers’ experience of working with carers and running carer groups. They covered: carers’ experiences and support needs; their experiences of the group and its impact; views on its structure and content; any helpful or challenging aspects. The topic guides were refined iteratively to include additional prompts that emerged as the interviews progressed.

IM conducted all semi-structured interviews in the period six weeks before and six weeks after the two group programmes ran. In 2021, telephone interviews were conducted due to coronavirus restrictions. In 2023, an option of face-to-face (at the community mental health team base) or telephone interview was offered. Interviews were audio-recorded and reflective notes were written by IM immediately after the interview. Recordings were transcribed and anonymised by IM and transferred to NVivo 9 (QSR International, Warrington, UK) software for analysis.

Information regarding participants’ age-range and carer role was also collected. GDPR prevented reporting of further demographic information to protect participants’ anonymity.

#### Reflective notes from group facilitators

Following the 2023 group, all four co-facilitators provided written reflections on: their thoughts on the group; their experience of facilitating it; what they observed carers took from it; any other comments. (Due to the challenges of running the group during the COVID-19 pandemic, these reflections were not collected following the 2021 group). These notes provided further data for triangulation with the qualitative data from the semi-structured interviews [35].

### Data analysis

A thematic approach to data analysis was selected in keeping with Braun and Clarke’s [36] view that this is appropriate to examine the perspectives of different participants and generate insightful findings [36]. There are various types of thematic analysis (e.g. codebook and reflexive) which vary according to their epistemological position (e.g. positivist vs constructivist) [37]. This study used reflexive thematic analysis (RTA). RTA operates within interpretivist and constructionist paradigms to enable richer interpretations of meaning, through creative and reflective engagement with the data [39]. Importantly for this study, RTA respects the subjectivity of participant experiences and embraces the subjectivity of the researcher [37]. The analysis was conducted inductively, so the study objectives were addressed directly from the people who experienced it (“bottom up”), rather than being shaped and constrained by previous theories or research (“top down”) [38]. Initial codes were therefore generated from the data itself. This constructivist, inductive approach tends to produce a richer description and deeper understanding of the data [38]. The coding was both semantic (identified through the explicit meaning of the data) and latent (identified through active interpretation to find hidden meanings and assumptions) [39]. In detail, the analysis process moved back and forth between these six stages iteratively and cyclically [36]:

1. IM immersed herself in the data by transcribing the interviews and reading the transcripts repeatedly, all the while searching for meaning, and keeping notes on key quotes, ideas for codes and initial interpretations [40].
2. Open coding (without a coding framework) was performed by systematically going through each of the transcripts, tagging key words or phrases with a descriptive “code” that captured their essence. IM kept reflexive notes throughout, to keep track of how thoughts and ideas for themes developed. 10% of the transcripts (*n* = 4) were analysed by two additional researchers (HW, JBa) to enhance the credibility of the analysis and bring a range of perspectives to the text [41, 42].
3. Candidate themes “patterns of shared meaning, cohering around a central concept” [43] were generated by: a) exploring the initial codes for connections, shared meaning or repetition, in order to collapse them into a single code/unit of analysis where possible b) active, reflexive and creative engagement with codes to construe patterns and relationships between them, “themes” [39]. The researchers (HW, IM, JBa) discussed their candidate themes until a consensus was reached.
4. Candidate themes were reviewed and refined by: a) IM considering whether the codes for each theme adequately support it and form a coherent pattern b) IM considering whether the themes interpret the overall dataset and answer the research question [36, 39].
5. Themes were defined and named.
6. Themes were further refined through the process of writing up the paper.

The same six step process was applied to the facilitators’ reflective notes.

### Reflexivity

In reflexive thematic analysis, the researcher is the instrument, using their own perspectives and beliefs as a resource [44]. The researcher’s awareness of these perspectives and how they may have influenced their interpretation of the data is vital [44].

IM had a dual role as the group’s creator and co-facilitator, and its researcher. She is a consultant psychiatrist who has worked with people with psychosis and their carers for many years. As a trainee, she had a formative experience when a patient, whose family had not been listened to by mental health services, died by suicide. She also has direct experience of how distressing it is to see a close relative slip into psychosis, and the challenges of accessing care. It is likely that her experiences influenced the study overall. IM’s prior knowledge of the relevant research may also have affected the analysis, although an inductive approach was used.

Jackie Bailey (JBa) and Henry Whittle (HW) brought their perspectives to the transcript analysis. HW is a trainee psychiatrist with an interest in relational psychiatry and experience of qualitative methods. JBa is a social worker, who has supported people with SMI for decades and previously ran a carer support group. Helen Killaspy (HK) is a senior clinical academic psychiatrist with specialist expertise in mental health rehabilitation who has led numerous national and international quantitative and qualitative studies. IM’s other co-supervisor, Jo Billings (JBi) is a Consultant Clinical Psychologist and experienced qualitative researcher, who has worked clinically with people with complex mental health needs and their families for over 23 years.

## Results

The characteristics of the 17 participants (ten in 2021, seven in 2023) are described in Table 3. In 2021, two participants dropped out of the group and did not complete the post-group interviews. There were no drop-outs in 2023. The interviews lasted 30–60 min and were conducted over the telephone, except two that were conducted face-to-face at the community team base.

**Table 3:** Characteristics of participants

Carer role	No. of carers	Age range	No. of carers
Mother	9	19–24	0
Father	3	25–34	3
Son	1	35–44	1
Daughter	1	45–54	2
Sister	2	55–64	3
Wife	1	65–74	6

The results are divided into two parts corresponding to the study objectives. Part 1 identified inductive themes and subthemes that addressed objective 1) ‘the impact of attending the group on participants’ experiences of caring’ (Table 4). The themes have been labelled to capture the dilemmas, dichotomies and developments in people’s experiences. This process was not linear and participants grappled with these issues over the course of the group. Part 2 identified inductive and deductive themes addressing objective 2) ‘views on the structure, content and processes of the group, to inform its ongoing development’ (Table 5).

**Table 4:** Study Objective 1—Themes and Sub-themes

Part One: Impact of attending the group on participants’ experiences of caring
**1. Isolated versus connected and supported**
1.1 Stigma
1.2 Lack of understanding and experience of psychosis within the general public
1.3 Social withdrawal amongst people with psychosis
1.4 Lack of support from mental health services
1.5 Restrictions on carers’ lives
*1.6 Impact of the group: connection and support*
**2. Disempowered versus empowered**
2.1 Lack of information about the illness
2.2 Excluded by services
*2.3 Impact of the group: empowerment: 2.31 “Feeling on the inside” 2.32 Information is power!*
**3. Guilt, grief and worry versus acceptance**
3.1 Guilt, grief and worry
*3.2 Impact of the group: acceptance*
**4. Unmet needs: the challenges associated with participants’ caring roles which were not addressed by group participation**
4.1 The need for individualised advice
4.2 The impact on the wider family

**Table 5:** Study objective 2—Themes

5. Part 2: Views on the group’s content and processes, and suggestions for its future development
5.1 Frequency, format and timing
5.2 Psychoeducational talks (content and delivery)
5.3 Small groups and their facilitation
5.4 Face-to-face versus online format
5.5 Recruitment and retention

All names have been changed to ensure anonymity.

### Part One: the impact of attending the group on participants’ experiences of caring

## Isolated versus connected and supported

### Stigma

Some participants reported that the stigma associated with schizophrenia and psychosis made them reluctant to seek support. One carer highlighted the greater stigma associated with mental illness compared to physical illness, manifesting in an absence of sympathy from friends when her relative was unwell.

*“My husband doesn’t really want people knowing and I’ve had to tell some people just for my sanity, just to be able to talk to people about the situation… so I’m kind of hiding a little bit about who I’m actually telling.” [Kelly].*

### Lack of understanding or experience of the condition within the general public

Many carers had found that the behaviour of the person they cared for was hard for people to relate to, and friends and wider family were unable to offer support or advice. Very few participants had received peer support or even met other carers of people with psychosis, adding to their sense of isolation.

*“…it’s actually really difficult, ‘cos it’s really hard for anyone who has had no experience to have any, absolutely any, understanding I would say. People can either start talking to him in a… or are kind of scared of him or talk to him in quite a patronising way when he is highly intelligent adult.” [Kelly].*

### Social withdrawal amongst people with psychosis

In some cases, participants carried the burden of caring alone, because their relative felt too paranoid or withdrawn to accept contact with anyone else.

*“…you know you can’t help Sally too much… she has been taking my help, but… she don’t talk to her sisters, she shuts them out. I’ve done it all these years on my own really, she don’t let her family in.” [Jackie].*

### A lack of support from mental health services

The majority of participants reported that the support provided by services had been inadequate and very few had previously attended a support group.

*“It’s been insufficient, and it’s felt a bit like we are on our own, having to kind of deal with it all… it’s been difficult, there’s been times when it has felt very difficult.” [Tony].*

### Restrictions on carers’ lives

Some carers reported that they socialised less due to their caring role, especially during acute relapses due to concern for their relative’s safety if left alone. At these times, carers felt burdened by responsibility for the welfare of a loved one behaving in a risky or unpredictable way and frustrated that services did little to support them or prevent their relatives’ deterioration.

*“So, we just kind of muddle through really… it puts a lot of restrictions on what we can do, ‘cos we don’t feel like we can leave Sarah on her own for very long… just kind of knowing that you’re on that pathway and just waiting for that bad thing to happen is very, very stressful and worrying.” [Tony].*

### Impact of the group: connection and support

For many participants, this was the first time they had met other carers of people with psychosis. Several participants described their relief at realising that their challenges were not unique. Shared experiences enabled carers to provide each other with empathy and validation. Participants found it helpful to have a dedicated space to talk openly about their caring role, particularly without their relative present.

*"Everyone is very empathetic… we all know how painful it is to see someone lose their mind and it’s, um, I think that’s a very strong thing for people to relate to each other with.” [Molly].*

Over time, as this sharing took place, some carers reported that they bonded and developed friendships, reducing their sense of isolation. This relationship building was also described in facilitators' reflective notes.

“*…the whole group was kind of—the way we became very intimate—we got to know each other… I grew fond of those people in my group… it kind of created a sense of community.” [Errol].*

However, it is important to note that some carers felt that other group members sometimes took up too much time sharing their experiences.

## Disempowered versus empowered

### Lack of information about the illness

Carers had rarely been given information about their relative’s diagnosis by mental health professionals. Instead, they had tried to research it themselves on the internet and in textbooks.

*“The trouble with using the internet and stuff like that… you have to go and research what that word means… it’s just difficult… better if they just explain to you in normal terms… I’d know more how to handle the situation.” [Sal].*

Without in-depth knowledge of symptoms and phenomenology, carers often struggled to understand their loved one’s experiences. They also lacked confidence in their ability to manage their relative and did not always know how to seek help. Overall, the lack of information significantly contributed to the stress of the caring role.

*“It took us a long time to realise what was going on because we weren’t really learning the symptoms and how psychosis really affected somebody.” [Sue]*

#### Excluded by services

Participants frequently reported that services did not listen to their concerns or seek their views. Carers often struggled to get their relatives help, only to find themselves excluded from care planning when services did step in.

*“I think that’s one of the things that has been very tough for carers that I’ve picked up over the years… is that they sometimes feel, you know, left carrying the can, but nobody really listens.” [Molly].*

For some, not knowing about their relative’s care was distressing. For others, the sense of powerlessness from being “on the outside” added to their burden. Those who felt most excluded viewed services negatively.

*“Honestly… honestly, it’s a little bit like, suddenly, it had become a closed institution.”* *[Kelly]*

## Impact of the group: empowerment

### Feeling “on the inside”

Several participants described how the group made them feel more valued by services, partly because senior professionals cared about their well-being and had given up their time to help. Some carers highlighted how meaningful it was to speak to professionals who listened to their concerns and were honest about failings in the system, rather than dismissing them. This validation from professionals built carers’ confidence. In some cases, attending the group improved carers’ attitudes towards services overall.

*“All the people in our group are people who have been carers for many, many years, so over time we have had lots of negative experiences, and so the ability to have that, to express that, to people who are in the system, to have it validated and heard… what I think was very good, that all of the three of you really wanted to hear what people felt.” [Erol].*

Furthermore, carers reported that their interactions with facilitators made them feel that psychiatrists were more approachable.

*“I thought it was wonderful that there were psychiatrists there. I really felt… valued as psychiatrists have always seemed… kind of remote and difficult to reach… And very nice to have the human face of psychiatry.*” *[Penny].*

Facilitators’ reflective notes also described positive relationship building across the carer/professional divide. They found that participants valued the opportunity to ask questions and discuss their views and experiences of services. They also noted the learning ran both ways: carers had unique insights into the struggles of people with SMI and into the challenges of working with the mental health system, having experienced the Trust’s many reconfigurations over decades.

### Information is power!

Participants valued the information provided (both tips from other carers and educational talks) and described ways in which this improved their experience of caring. For some, a greater understanding of their relative’s behaviour led to more empathic responses.

*“When this was going on I had no understanding of delusions but now I understand exactly what happened there you know… and that’s very helpful.” [Molly]*

*“…it’s so useful to be reminded about all these things, about all these symptoms and also to be reminded that the illness means they just what to spent lots of time alone… they want to be alone and quiet and not doing anything particularly… which to us seems a nightmare.” [Gavin].*

Other participants described how they could apply the information provided to better support their relative.

*“The occupational therapy session… that was to me the one that offered the most new tools.” [Erol]*

Information about medication was also valued.

*“In terms of… supporting him to make a decision around clozapine or not will have made a massive difference, it was a very big influence. So, there’s some really tangible impact actually.” [Kelly].*

One carer described how understanding side effects meant they responded more empathetically to their relative’s weight gain.

Most participants found the group helped them to be more aware of the support they and their relatives were entitled to and enabled them to ask for it.

*“…I think it’s given me more confidence in knowing what I could ask for. Now that I know what is out there, I know what I can ask for, when you don’t know, you don’t know to bring to up or who to ask for it.” [Sal].*

However, two carers found it frustrating to hear about ‘ideal world’ services which were not actually available to them.

“*So, we seem to be having two stories… one of the theory and what we are learning about and one of practice… acknowledging the gulf between the theory and practice.” [Sue].*

## Grief, guilt and worry versus acceptance

### Grief, guilt and worry

Group members who were parents tended to blame themselves for their child’s illness.

“*You know every parent with a child with mental health issues is being judged. Because it’s… it’s where, why did they go wrong kind of thing… I know that, um, I must have not handled it in the best way, you know I could have.” [Lucy].*

Parents described the pain of losing the person their child was before they became unwell, which one mother described as a bereavement. They also described relinquishing the hopes they had held for their child and felt worried about their child’s future, particularly as they aged.

*“…I suppose just dealing with your own feelings about it, because you are sort of grieving for what you had hoped for, for her and her life, and then you sort of grieve about the fact that that isn’t actually going to happen…” [Tony].*

One sibling carer reported that she attended the group to prepare to take over the caring role from her ageing parents.

### Impact of the group: acceptance and stepping back

Some parents described how hearing the insights and experiences of other parents helped them accept their child’s situation. One parent described this as a journey and reported feeling “invigorated” by the group.

***“*** *Hearing from other people on how they were struggling to come to a point of acceptance about it as opposed to anger about it, or guilt about it, or other emotions about it… so that was to me a very special to have other people talk about their journey, and how we all now seem to have arrived at this point of ‘oh it doesn’t throw us again anymore’, ‘it doesn’t upset… the absolute emotional turmoil is kind of settling.” [Errol].*

For one family, this acceptance came with a newfound ability to step back; to give more time to themselves and intervene less with their child. This reduced their burden and helped their son, too:

*“I just feel that there has been a subtle but very important shift in the way we manage our caring role and how we feel about our son and his illness… I think we’ve become more understanding and not so much pushing for him to get better… we have to accept him where he is… having kept our distance while doing lots of things with him… but not asking questions or making suggestions there has been a really noticeable improvement in his… you know how he is.” [Norma].*

## Unmet needs: the challenges associated with participants’ caring roles which were not addressed by group participation

### The need for individualised advice

Carers frequently requested personalised advice to help them manage the behaviours and issues specific to their relative. Unfortunately, this was not possible in a group format.

*“I mean I suppose you guys aren’t really in a position to really advise on individual cases and things, I’m just thinking of some of the things that people were going through and I feel like did that feel unresolved.” [Annie].*

### The impact of the illness on the wider family

Several carers spoke of the significant impact their relative’s illness had on the whole family unit.

*“…well as a result of all the illness actually, the family has been somewhat torn apart in terms of relationships. So, all of that family stuff is very hard to deal with.” [Tony]*

One participant commented that the impact on the wider family was not discussed sufficiently within the group. Another described how group participation brought up difficult issues for the family and led to disagreements.

*“I didn’t kind of anticipate that it may kind of you know be triggering… there are definitely things about my brother that we don’t agree on, like his future… what we imagine for him in his future, and we ended up having quite a big you know disagreement about that which was really tough.” [Serena].*

### Part 2: Carer and faciliator views on the group’s structure, content and processes, and suggestions for its future development

Overall, the group received very positive feedback and all carers said they would recommend it to friends or family. All facilitators valued the experience of facilitating the group and found it highly rewarding.

“…*brilliant, powerful. One of the most rewarding and useful parts of my work… I always learn so much.” [Dr Smith—facilitator].*

### Frequency, format and timing

The group’s structure, content and processes generally received positive feedback.

*“I thought it worked much better than I thought it was going to work.” [Molly]*

The evening timing was favoured by most participants due to work or daytime commitments. The weekly frequency was acceptable to most, but two carers would have preferred two-weekly, and many were open to this suggestion.

*“Oh I like weekly, cos I think if it fortnightly it sort of.. it’s too long a gap I think personally” [Molly]*

Several participants stated that the group could have been extended from 8 to 10 weeks, and many expressed regret at the loss of contact with peers once it ended. All supported a plan to run monthly ‘catch up’ sessions.

*“I think follow ups would be good, just to keep that link going, I think that’s a good idea*” *[Sue].*

Carers said they valued knowing the purpose and format of the group beforehand, and facilitators reflected that this knowledge improved engagement. Facilitators reported that they found it feasible to set up and facilitate the group. However, they noted that they gave up their own time to do this.

### Psychoeducational talks (content and delivery)

The psychoeducational talks were perceived as helpful by all participants and the content was generally considered appropriate and well-pitched.

*“All the presentations were super helpful… I can look back at them… explaining what different health professionals do, so you know what different medication is, or symptoms…” [Serena].*

However, some carers found the information familiar (but helpful to hear again) and wanted to know more about the latest advances in treatment, while others found some talks too complicated.

Suggestions for changes to content included advice on managing stress and self-care, and more signposting to community resources. Carers of people with bipolar affective disorder found the talks too focussed on schizophrenia. Many participants said they particularly valued access to senior professionals during question time and wished this could have been longer. One carer suggested the talks be available online to watch beforehand, to save time in the group itself, however two others considered this an unrealistic demand on carers’ time. Participants found it helpful to receive handouts of the presentations beforehand.

### Small groups and their facilitation

Many participants would have preferred longer in the small groups, although some commented that their size (6 carers on average) generally allowed time for everyone to speak and facilitated relationship building. The similarity of carers’ experiences was also identified as key to the small group’s effectiveness. Respondents highlighted some key characteristics of the group facilitators including warmth, honesty and dedication to helping carers (“not just part of the job”). Having a flexible approach to facilitation was also noted, including using questions, prompts and exercises to encourage engagement.

The main challenge to the success of the small groups identified by participants was that some carers tended to dominate the sessions. One carer suggested facilitators receive training in group facilitation to help manage this. Facilitators’ reflective notes acknowledged similar challenges, including how to ensure all voices were heard, keeping the sessions to time, and responding to the difficult issues and emotions brought up.

### Face-to-face versus on-line format

While some carers acknowledged online delivery was convenient, the vast majority reported they preferred face-to-face sessions. Participants of the 2023 ‘in-person’ group all stated that they appreciated building relationships face-to-face, and none supported a switch to online delivery. They also noted that they valued the informal time for conversations during the face-to-face tea break.

Facilitators with experience of both delivery modes commented that the online group was more successful than they expected, with carers still able to share difficult experiences, but that relationship building was stronger face-to-face.

*“It’s very difficult to connect on screen and a lot of this is about connection and support… I mean I would say in person massively preferable. It’s maybe easier for people to make a meeting online, but you don’t get the chat in the break—I don’t think you’ll get relationships in the same way.” [Kelly].*

### Recruitment and retention

Facilitators’ reflective notes indicated that the group’s recruitment strategy was inadequate. Many carers were not invited as staff tended only to invite ‘well-known’ or ‘suitable’ carers, rather than all carers. In particular, carers from BME groups were under-represented. There were very few drop-outs from the group (2021 = 2, 2023 = 1).

## Discussion

This service evaluation aimed to investigate the impact of psychoeducation and support groups for carers of people with SMI, and to gather feedback on the structure, content and processes of the group to inform its further development. Our study corroborated existing evidence that such groups are beneficial for carers of people with SMI, provided evidence of their potential benefit to carers of people with longer term psychosis, and informed refinements to the group design.

### The impact of connecting carers with each other

Carers of people with psychosis report up to 10 times the level of isolation of non-carers [5]. Our findings concurred with previous studies in identifying social isolation as a major issue [20, 19, 45]. We also identified insights into its potential causes, including: stigma and a lack of understanding about SMI among carers’ social networks; the social withdrawal of the patient; a lack of support from services; and a perceived need for close supervision of their relative due to concerns about risk. Some of these factors are specific to psychosis, highlighting the need for focused support for this carer group. In common with previous studies, the carers’ group enabled participants to share experiences and build friendships which reduced this sense of isolation [19, 20, 30] and, again, in keeping with previous research, carers welcomed the opportunity to talk freely, without the service user present [20].

### The impact of connecting carers with services

It is well established that carers of people with psychosis are not included adequately by mental health services [45, 29]. Our results illustrate the negative consequences of this. Carers reported feeling unheard, unappreciated and unsupported by services, and distressed at being ‘shut out’. By contrast, the group provided an opportunity for services to include and value carers. This fostered better relationships between carers and professionals, and, as other studies have found, appeared to empower carers to consider asking for greater involvement and better support for them and their relative [20, 30]. A reciprocal beneficial impact was reported by facilitators, who learnt from carers and valued the improved connection highly. Our findings emphasise the need for initiatives like the Triangle of Care which seek to improve practice towards carers by mental health services [46].

### The impact of psychoeducation

Similar to previous studies, our results illustrate the need to provide carers with information about their relative’s illness [13, 29]. Carers described the stress of managing their relative with little understanding of symptoms or how to help, and their struggle to inform themselves. Consistent with the existing literature, participants found the psychoeducational component of our group helpful, and there were suggestions of benefits to the patient too [31, 30, 20, 19]. Carers learnt from each other, as well as from the educational talks and found the information improved their empathy and reduced feelings of impotence.

### Carers have different needs at different stages in their journey

Previous studies of parents of children with psychosis describe the emotional burden of the situation, including feelings of grief, self-blame and worry about the future [45, 20, 19]. Many of our participants described similar experiences and worry about the future was prominent, perhaps reflecting the large proportion of ageing parents in our groups. We found that group attendance helped parents progress from denial towards acceptance of their child’s psychosis, a psychological journey described by Kuipers et al. 2018, who noted this acceptance improved the patient-carer relationship [47]. We also identified support needs for siblings of people with SMI—an under-researched group, often overlooked by services [31]. Mental health professionals working with people with longer term problems need to better consider the issues faced by older parents and sibling carers. Our results also illustrated that carers value more advanced information on treatment options as their relative’s illness progresses.

### Key components of the group’s structure, processes and content

We delivered two series of groups for carers with a low drop-out rate, suggesting they were both feasible to implement and acceptable to carers. Key components of the group’s processes, structure and content included: providing information on the group’s purpose and structure beforehand; having carers with similar experiences; limiting participant numbers for small-group work; involving senior professionals including psychiatrists; question/discussion time with professionals; warmth, dedication and openness of facilitators.

There were some suggestions for the group’s refinement, including more sessions, more skilled facilitation (by senior and/or trained staff), and minor adjustments to content of the talks. Improving the ‘recruitment strategy’ and accessibility to carers from BME groups was also noted. Staff would be more likely to refer carers if the Trust implemented ‘carer awareness’ strategies (e.g. training, carer ‘champions’) as recommended by Triangle of Care guidance [46].

### Strengths

This study provided much-needed evidence to inform the design of an optimal group-based psychoeducation and support intervention for carers of people with psychosis. It also addressed a gap in the literature by focussing on carers of people with longer term SMI. The use of an in-depth qualitative approach facilitated detailed feedback on the group’s structure, content and processes, and a nuanced exploration of its helpful aspects and areas for improvement. This provided a better understanding of the components and format likely to benefit carers most. The study also contributes to the limited literature on the experiences of carers of people with longer term psychosis [45]. The triangulation of data from carer interviews with group facilitator reflective notes is a further strength of our design and provided corroboration for many of the themes we identified. Data analysis followed a well-established approach and 10% of the transcripts were coded by an additional researcher to enhance credibility and bring a range of perspectives to the findings.

### Limitations

Our sample was small, lacked ethnic diversity and consisted mainly of parents. This limits the transferability of the findings to the wider population of carers. In addition, around one third of the group attendees declined to participate in the evaluation and response/non-response bias is therefore acknowledged. Although it is reassuring that the demographics did not differ between participants and non-participants, it is possible that participation altered the group experience itself (i.e. through better awareness of the group’s purpose and closer ties with the facilitator who completed the interviews). The role of the first author (IM) in both leading the group and its evaluation can be seen as a strength but also introduced limitations. Participants may have felt reticent about sharing negative views with IM, but conversely, the rapport developed may have facilitated greater openness. IM’s analysis of the transcripts and reflexivity will also have been influenced by her dual roles. Finally, only carers who attended the group participated in the evaluation, and therefore there was no exploration of group acceptability, or barriers to participation, for carers who did not attend.

### Implications for practice, policy and future research

The need to support carers is already embedded in policy and practice guidelines. The challenge is how to implement this. The findings of this study are encouraging and demonstrate that carer groups are a feasible way to provide carers with psychoeducation and support in a busy, inner city mental health service. These groups also met some needs unmet by structured family interventions, such as psychoeducation across a wide range of topics and the opportunity to talk openly with other carers without the service user present. However, it is important to note that the group could not meet carers’ need for individualised advice and systemic therapy, and could not replace evidence-based, structured family interventions. Rather, carer groups may provide a useful alternative where family interventions are unavailable or unsuitable. Our results also emphasise the need for a change in practice towards carers by mental health professionals.

The quantitative evidence for carer support groups in psychosis is weak and there are no UK based trials of face-to-face groups. Recent research has focussed on digital psychoeducation interventions for carers [26, 25]. Our results, which highlight the importance of face-to-face contact and relationship building, may help to explain why these studies have failed to show positive results. Further RCTs of face-to-face carer support group interventions are needed, perhaps using on-line psychoeducational materials. This would lessen the workload for group facilitators and provide more time for interaction and small group discussion. Future research into carer support groups should investigate: variations to their format and content; their impact on carer and patient outcomes; their cost-effectiveness; the role of peer facilitators; how to improve recruitment and accessibility for carers from minority groups; and barriers to implementation.

## Conclusions

Our study evaluated a psychoeducation and support group for carers of people with SMI. It showed that the intervention had a feasible and acceptable format and structure, which flattened the carer-provider hierarchy, and enabled carers to share experiences and gain knowledge and confidence, all of which they found beneficial. The results may explain why recent RCTs that have relied on digital contact have not proven to be effective. Further research is needed to establish the clinical and cost-effectiveness of carer support groups, and their role as additions or alternatives to established family interventions.

## Supplementary Information

Supplementary Material 1.

## Data Availability

The data that support the findings of this study are not publicly available to protect anonymity, but are available on request from the corresponding author (IM).

## Abbreviations

SMI: Serious mental illness
FI: Family interventions
RTA: Reflexive thematic analysis
RCT: Randomised controlled trial
BME: Black and minority ethnic
HRA: Health research authority
GDPR: General Data Protection Regulations
R&D: Research and development
