# Supplementary material for: Experiences of participation in an education and support group for carers of people with longer-term psychosis: a qualitative study
Source: BMC Psychol. 2025 Feb 21;13:153. doi: 10.1186/s40359-024-02288-2 (PMC11846203; doi:10.1186/s40359-024-02288-2)
Supplement: Supplementary file 1 — Supplementary Material 1. [file 40359_2024_2288_MOESM1_ESM.docx]

**
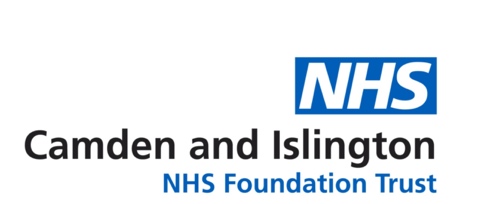
Appendix 1**

**Interview Topic Guide^^[[1]](#footnote-1)^^: Pre-group Interview – All Participants**

**A service evaluation of an education and support group for carers of people with psychosis and schizophrenia.**

**Introduction**

Thank you so much for giving up your time today. My name is Issy Millard. I’m a trainee psychiatrist in Camden and Islington Trust and I’ll also be facilitating the group. As it says in the information sheet, I’ll be asking you today about your views on the plans for the carers’ group, and about your experiences as a carer.

Just to reiterate a few points about the session:

- It will last about 30 minutes and will be recorded – this allows us to capture your views as best we can.
- Everything discussed in this session will be completely anonymous and confidential, unless evidence of wrongdoing or potential harm is disclosed. In this situation, I may need to pass this information on to the relevant statutory authorities / agency – I would discuss this with you first.
- You can stop the interview at any time.
- if there are any questions you would rather not answer just let me know and we will move on to the next one.

- Before the audio-recording starts, I would like to ask for information on your age range and carer role. This will be recorded in writing, anonymously to help us understand whether the carers who participate in the study are representative of the wider carer population.

Are you: 18-24/ 25-34/ 35-44/ 45-54/ 55-64/ 65-74

What is your relationship to the person you care for: parent/sibling/spouse or partner/child/other relative/friend.

**Part A: To start off with, it would be helpful for us to learn a bit about what support or education you may have been offered in the past:**

1. What, if anything, has your family been offered so far? Who was it provided by? Did you find it helpful?
2. Have you received any information about psychosis or schizophrenia from a mental health professional in the past?
3. Have you received any support from other carers in the past?
4. How do you feel about the support you’ve been offered so far with your caring role?

**Part B: The aim of this part of the interview is to hear about your hopes for the group, and to gather your views on its structure and content. I’m going to start by briefly describing our current plan for how the group will run so you can see whether you think any changes need to be made:**

There will be up to sixteen carers in the group and it will run in the evenings from 5:30-7:30pm. It will be facilitated by two mental health professionals.

The idea is that the group provides you with information about psychosis, as well as support with your caring role. To do this we are planning to start the sessions off with a half an hour talk about various aspects of psychosis, with time for questions at the end. The talks will include information about diagnosis and symptoms, medication, psychological interventions, navigating mental health services and the Mental Health Act. They will be delivered by a mixture of psychiatrists, social workers and a psychologist.

We’ll then have a short break. After the break carers will form small groups of five. You’ll be in the same group each week so that you all get to know each other. The idea behind these groups is that you get to meet other carers in similar situations – and we hope that you’ll all be able to share experiences and coping strategies and support each other.

1. Is this what you were expecting from the group? If not, is there anything else you were hoping for?
2. Do you have any suggestions or comments on the structure I’ve described? Is there anything you think won’t work well for you or that should be changed?
3. Do you have any concerns about the group being online?
4. Are there any topics that you would like to be included in the talks that I haven’t mentioned?

**Part C: In this section, we would like to understand more about the difficulties that carers may experience in their caring role:**

1. What are the main problems (if any) you have as a result of your caring role that you would like help with?
2. What impact do you think the illness has had on your friendships and the amount you go out socially? Do you feel isolated sometimes?
3. What has your experience been of using mental health services? Do you feel confident about accessing mental health services or seeking help for your relative when they need it?
4. Do you feel well-informed about your relative’s illness? If not, does this make things more difficult for you?

**
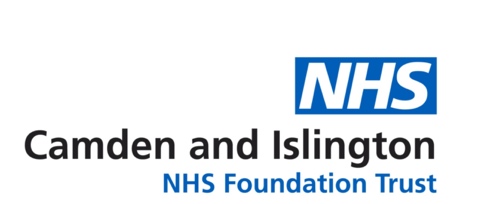
**

**Interview Topic Guide^^[[2]](#footnote-2)^^: Post-group Interview – All Participants**

**A service evaluation of an education and support group for carers of people with psychosis and schizophrenia.**

**Introduction**

Thank you so much for giving up your time to speak to me again today. As it says in the information sheet, I’ll be asking you today about your experience of the carers’ group and any impact it may have had on your role as a carer.

Just to reiterate a few points about the session -

- The session will last about 30 minutes and will be recorded – this allows us to capture your views as best we can.
- Everything discussed in this session will be completely anonymous and confidential, unless evidence of wrongdoing or potential harm is disclosed. In this situation, I may need to pass this information on to the relevant statutory authorities / agency – I would discuss this with you first.
- You can stop the interview at any time.
- If there are any questions you would rather not answer just let me know and we will move on to the next one.

**Part A: I’ll start by asking you some questions about your experience of the group. We’re keen to hear your feedback so we can improve it for next time:**

1. What was particularly helpful about attending the group?
2. Was anything unhelpful?
3. Regarding the educational talks:

- did they provide the right depth of information.. was it too much information or too little?
- were they easy to follow?
- did they cover the right topics? Is there anything we should include (or not include) next time?
- would you have preferred more time on educational talks? Or less?
- do you have any other comments on how we could improve these?

1. Regarding the small groups:

- how did you find talking with other carers about your experiences?
- how did you find hearing about other carers’ experiences?
- did you find the group exercises helpful?
- did you feel supported by other carers?
- were you able to discuss the things you wanted to?
- could anything have improved the small group sessions?

1. would you have preferred more time in small groups? Or less? Was it helpful having a mental health professional facilitate the group?
2. How would you feel about the group being online?
3. Was the evening timing of the sessions suitable for you or would another time be better?
4. Did you have enough sessions and were they long enough?
5. Overall, could anything else have improved the group?
6. How likely are you to recommend the group to other carers?

**Part B: We would like to understand whether the group has had any impact your experience as a carer:**

1. To what extent have any difficulties you experience in your role as a carer been helped by the group?
2. Do you feel the group has helped you to feel more connected? If yes, in what way…
3. Do you feel it has helped you feel more confident about accessing mental health services or seeking help for your relative when they need it? If yes, in what way…
4. Do you feel it has helped you understand more about your relative’s illness? If yes, in what way…

1. The 2021 pre -group interview topic guide varied slightly as the sessions were online. [↑](#footnote-ref-1)
2. The 2021 post-group topic guide varied slightly as the sessions were online. [↑](#footnote-ref-2)
